# The effect of different doses of estradiol benzoate on follicle development and twin birth in beef cattle with induced superovulation

**DOI:** 10.5194/aab-67-533-2024

**Published:** 2024-11-08

**Authors:** Weibin Zeng, Lei An, Yanping Wang, Xinli Gu, Yusheng Qin, Jianhui Tian

**Affiliations:** 1 College of Animal Science and Technology, Shihezi University, 832003, Shihezi, China; 2 College of Animal Science and Technology, China Agricultural University, 100193, Beijing, China

## Abstract

The aim of this study was to evaluate the effect of different doses of estradiol benzoate (EB) on inhibiting the development of non-dominant follicles and inducing twin calves in beef heifers. Beef heifers were synchronized using estradiol plus progesterone (P4), and superovulation was induced using a small-dose follicle-stimulating hormone (FSH) protocol. From day 6.5 to day 7.5, every heifer was treated with 0, 0.1, 0.2, or 0.5 mg EB three times at 12 h intervals to eliminate excess dominant follicles. The diameters of the two largest follicles (F1 and F2) continually increased from day 3.5 to day 10. However, the growth rate was constrained by exogenous EB, and the degree of suppression was greatest in the 0.5 mg EB treatment compared with other treatments. As a result, the number of large follicles ( ≥10 mm) decreased as the EB dose increased. Compared with the control treatment, the incidence of animals experiencing triple ovulation was significantly lower in the 0.5 mg EB treatment; however, the single-ovulation rate showed the inverse. This study demonstrated that 0.2 mg EB could modulate the development of two to three co-dominant follicles after a small-dose FSH treatment. The number of twin births was higher in the 0.2 mg EB treatment.

## 1 Introduction

Cattle are monovular and naturally single-birth animals, producing only one calf per year. Dairy cows present an overall twinning rate of 3.01 %, which has been related to parity, heredity, and season (Nejati-Javaremi et al., 2009). It has been reported that the percentages of double ovulation for animals experiencing their first, second, and third or more lactations are 6.7 %, 16.6 %, and 25 %, respectively (Fricke and Wiltbank, 1999). The twinning rate is lower in beef cattle than in dairy cows, with rates of 0.6 % for American Hereford and 5 % for French Maine-Anjou and Holstein beef cattle (Vinet et al., 2012). To improve the reproductive efficiency of cattle, various methods have been implemented to control the number of dominant follicles in the ovary, form co-dominant follicles, and result in the subsequent ovulation of two follicles, thereby achieving the production of two calves from a single birth. Such methods include genetic selection, embryo transfer, hormone immunization, and follicular ablation (Guerra-Martinez et al., 1990; Morris et al., 1997; Echternkamp et al., 2004, 2007; Mussard et al., 2007). Large exogenous doses of follicle-stimulating hormone (FSH) are used to induce superovulation; moreover, small doses of FSH have been used to increase the incidence of co-dominant follicles (Davis and Bishop, 1992; Murphy et al., 1998) or double or triple ovulation in cattle (Palhao et al., 2009; Glick et al., 2013). Although the simultaneous ovulation of multiple follicles could increase the cattle production rate, the implantation of more than three embryos will lead to pregnancy loss (Çobanoğlu et al., 2010). Therefore, it is critical to precisely control the number of ovulated follicles.

A follicular wave on the bovine ovary was produced via the stimulation of a large amount of FSH. The peak FSH value appeared when the diameter of the largest follicle (F1) reached 4 mm (Ginther et al., 1996), at which stage follicles enter the co-growth stage. The concentration of FSH then began to decrease and reached the lowest concentration at 10–24 h after follicular dominance (Ginther et al., 1999, 2000b). At the beginning of follicular dominance, only larger follicles can use the lower concentration of FSH to continue growth, whereas smaller follicles are unable use it (Bergfelt et al., 2000). As a result, the larger follicles became ovulated follicles, whereas the smaller follicles became atretic follicles (Ginther et al., 2000a). Compared with animals with a single dominant follicle, cattle with two dominant follicles had a higher amount of FSH in serum before follicle dominance, although the FSH decreased more rapidly after dominance (Lopez et al., 2005b). During the preovulatory period in heifers that ovulate from two (compared with one) follicles, circulating concentrations of estradiol-17 β were greater, the diameter of follicles and the concentration of FSH were reduced, and the luteinizing hormone (LH) surge occurred sooner (Palhao et al., 2010). Lower FSH amounts after follicular dominance were related to a higher oestradiol (E2) concentration. A negative effect of estradiol benzoate (EB) on FSH levels in lactating women in the third week postpartum has been observed: FSH levels were elevated by the third week postpartum and decreased 24 h after EB administration (Elías et al., 1981). A similar relationship between FSH and E2 has also been confirmed with respect to double ovulation in horses (Ginther et al., 2008, 2009).

EB is a kind of oestrogen medicine. EB–P4 treatment could induce the emergence of a new follicular wave (Taniguchi et al., 2007). The administration of EB has been shown to induce atresia of the dominant follicle in the ovaries of cattle within 36 h, but the emergence of a new wave of follicular development was observed to be delayed by 3–5 d (Burke et al., 2003). EB has the same pharmacodynamic effects as E2. Plasma FSH concentrations have been shown to decrease by 12 h after oestradiol or EB treatment in all beef cattle, reaching a minimum at 24 h post-treatment (Martinez et al., 2005). Therefore, the present study aimed to compare the effects of different doses of EB on inducing twin production in beef cattle.

## 2 Materials and methods

### 2.1 Experimental animals

This experiment used 190 Simmental beef heifers (age: 18–20 months; weight: 600–650 kg). All heifers had regular estrus cycles and ovulation. The study was conducted in autumn. The heifers were held in open stalls at Tianhe cattle farm, Shijiazhuang, China. They were fed silage or hay supplemented with 2–3 kg of corn flour ad libitum and given free access to water. Animals were handled in accordance with the Chinese Department of Agriculture's Guide for the Care and Use of Agricultural Animals in Research.

**Figure 1:**
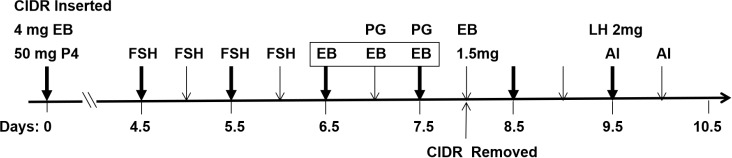
Diagram of the experimental protocol.

### 2.2 Experimental protocol

The beef heifers were treated according to the flow diagram shown in Fig. 1. On day 0, a controlled internal drug release (CIDR) with 1.5 g P4 (US Pfizer, New York, USA) 130 was inserted, and intramuscular injections of 4 mg of EB (Sansheng Biological Technology Co., Ltd., Ningbo, China) and 50 mg of P4 (Sansheng Biological Technology Co., Ltd., Ningbo, China) were carried out. From day 4.5 to day 6, FSH (SIOUX Biochemical Inc., IA, USA) was injected intramuscularly four times at 12 h intervals at doses of 6, 6, 5, and 5 U (where U denotes unit) per head. From day 6.5 to day 7.5, all beef heifers were randomly assigned to one of four groups according to the following treatments: different intramuscular doses of EB per head (0.1 mg, n=50 ; 0.2 mg, n=60 ; 0.5 mg, n=40 ) or saline (control, n=40 ). These treatments were administered three times at 12 h intervals. Cloprostenol (PG, Sansheng Biological Technology Co., Ltd., Ningbo, China) was also injected intramuscularly at a dose of 0.4 mg per head the last two times. On day 8, the CIDR was removed, and 1.5 mg of EB was injected intramuscularly. On day 9.5 and day 10, artificial insemination (AI) was performed using common frozen–thawed semen from the same Simmental bull, and 2 mg of LH (Sansheng Biological Technology Co., Ltd., Ningbo, China) was injected intramuscularly during the first AI.

### 2.3 Dynamics of follicular development

A total of 10 Simmental cattle were randomly selected from each EB treatment group (resulting in 40 animals overall) to monitor the dynamics of follicular development via transrectal ultrasonography (7.5 MHz linear array; HS-2000, Honda Electronics, Japan). The diameter and number of follicles were measured twice daily from day 3.5 until ovulation. The two largest follicles (F1 and F2; F1 was the largest follicle and F2 was the second-largest follicle) were defined according to the follicle diameter. Follicles with a diameter of ≥10 mm were classified as ovulatory-sized (large) follicles (Sartori et al., 2001), whereas those with a diameter of 6–9.9 mm were classified as medium-sized follicles (Gimenes et al., 2008).

### 2.4 Estrus, ovulation, and pregnancy

Behavioural estrus of 190 heifers was monitored visually four times daily for 2 d after removing the CIDR. Estrus was detected by visual observation, including standing heat, mounting other heifers, sniffing the vagina of other heifers, swelling or hyperemia of the vagina, and mucous from the vagina. Transrectal ultrasound examinations were carried out on day 7 post-AI to assess ovulation and the presence of corpus luteum (CL). Ovulation was defined as the presence of a recently formed CL, and the number of follicles ovulated was confirmed by the number of CL in the two ovaries. Conception was diagnosed by ultrasonography via the detection of a viable embryo on day 42 post-AI.

### 2.5 Estradiol concentration assays

To examine whether exogenous EB administration can modulate the E2 levels, we measured the E2 dynamic changes in the serum of heifers. Before each instance of monitoring follicular development by transrectal ultrasonography, blood samples were collected from the tail veins of the 40 originally selected heifers. Serum was separated by centrifugation ( 1500×g for 10 min) at room temperature after standing for 3 h at 4° and was then stored at -20° until the hormonal assay was carried out. The E2 concentration of each sample was measured using an ultrasensitive radioimmunoassay (RIA) kit (DSL-4800, Diagnostic Systems Laboratories, Webster, TX). The standard curve was prepared with charcoal-stripped bovine plasma (Turzillo and Fortune, 1990). Cross-reactivity of the assay was 0.4 % for estriol and 2.4 % for estrone. The minimal detectable amount was 0.5 pg mL^−1^, and the intra-assay and inter-assay coefficient of variation (CV) values were 3 % and 5 %, respectively.

### 2.6 Statistical analysis

The experimental model included the experimental treatments, the heifers within treatments, the days, and the day–treatment interaction. Categorical data (e.g. follicular diameter, follicular number, estrus, and ovulation) were all analysed using the least significant difference (LSD) test. The effects of treatment, time, and the treatment–time interaction on concentrations of E2 were analysed using an ANOVA with a repeated measures analysis included in the model (Burke et al., 2007). Single-point or discrete data were analysed using a one-way ANOVA. Specific mean comparisons were made using the SLICE program in the SAS software following detection of a significant effect in the main model. Data were presented as the least-squares means and the standard error of the mean. Significance was set at a 5 % level.

**Figure 2:**
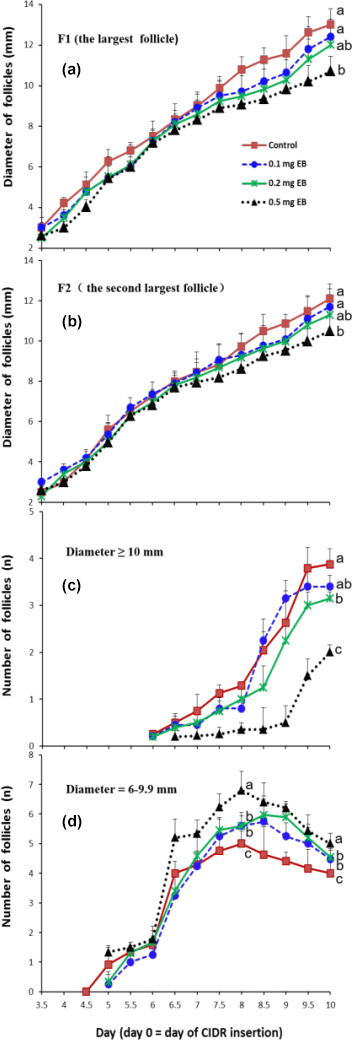
Effects of different doses of EB on the development of F1 (the largest follicle; **a**), F2 (the second-largest follicle; **b**), and the number of large follicles (diameter ≥10 mm; **c**) and medium follicles (diameter =6 –9.9 mm; **d**).

## 3 Results and analysis

### 3.1 Developmental dynamics of the largest follicle (F1) and the second-largest follicle (F2)

The effects of EB on the diameter of the largest follicle (F1) and the second-largest follicle (F2) are presented in Fig. 2a and b, respectively. The diameters of the two largest follicles did not show a significant difference among treatments from day 3.5 to day 6.5. However, the follicles' growth rate decreased as the dose of EB increased after day 6.5. Compared with the control, the 0.5 mg EB treatment presented smaller diameters for F1 (control =13 mm vs. 0.5 EB =10.7 mm ) and F2 (control =12.1 mm vs. 0.5 EB =10.5 mm) on day 10. The diameters of the 0.1 and 0.2 mg EB treatments did not differ from the control values.

### 3.2 Changes in the number of large follicles and medium-sized follicles

The number of large follicles (diameter ≥10 mm) decreased as the EB dosage increased (Fig. 2c), resulting in an average of 2.00, 3.15, and 3.88 follicles in the 0.5 mg EB treatment group, the 0.2 mg EB treatment group, and the control group, respectively, on day 10. However the 0.1 mg EB treatment (3.4 follicles) did not show a difference compared with the control group. The number of medium-sized (diameter =6 –9.9 mm) follicles increased significantly from day 6 to day 6.5 (Fig. 2d); in contrast, their numbers declined from day 8 to day 10, as some medium-sized follicles developed into large follicles or atretic follicles, and their diameters were then no longer in the aforementioned range of medium-sized follicles.

**Table 1:** Effect of different doses of EB on estrus, ovulation, and pregnancy in beef heifers.

Item	Control	Treatment 1	Treatment 2	Treatment 3
		(0.1 mg EB)	(0.2 mg EB)	(0.5 mg EB)
No. of exposed beef heifers	40	50	60	40
Estrus rate (%)^1^	95.0 (38/40)	96.0 (48/50)	93.3 (56/60)	85.0 (34/40)
Ovulation				
Ovulation rate (%)^2^	95.0 (38/40)	96.0 (48/50)	91.6 (55/60)	80.0 (32/40)
Single ovulation (%)^3^	13.2 (5/38)^a^	12.5 (6/48)^a^	10.9 (6/55)^a^	34.3 (11/32)^b^
Double/triple ovulation (%)^3^	57.9 (22/38)^a,c^	62.5 (30/48)^a,b^	80.0 (44/55)^b^	40.6 (13/32)^c^
More than triple ovulation (%)^3^	28.9 (11/38)^a^	25.0 (12/48)^a,b^	9.1 (5/55)^b^	6.2 (2/32)^b^
Pregnancy rate on day 42 (%)^4^	89.5 (34/38)	89.6 (43/48)	90.9 (50/55)	84.3 (27/32)
Twin-birth rate (%)^5^	35.3 (12/34)^a^	37.2 (16/43)^a^	58.0 (29/50)^b^	29.6 (8/27)^a^

^a–d^ Different letters within the same row indicate a signficant difference between groups ( P<0.05 ). ^1^ Number of heifers with estrus performance divided by the total number of animal enrolled in the study. ^2^ Number of heifers with one or more new CL formed 7 d post-AI divided by the total number of animal enrolled in the study. ^3^ Number of heifers with one or more new CL formed 7 d post-AI divided by the number of animals that had ovulated. ^4^ Number of heifers with a viable embryo 42 d post-AI divided by the number of animals that had ovulated. ^5^ Number of heifers giving birth to twin calves divided by the number of pregnant animals. This did not include dead fetuses. When an animal had difficulty giving birth, assistance was provided.

### 3.3 Effect of EB treatment on estrus, ovulation, and pregnancy

There was no influence of EB treatment on estrus, ovulation, or pregnancy (Table 1). Compared with the control, the 0.5 mg EB treatment induced a lower rate of animals with more than triple ovulation (control =28.9% vs. 0.5 EB =6.2% ; P<0.05 ) and a greater rate of animals displaying single ovulation (control =13.2% vs. 0.5 EB =34.3% ; P<0.05 ). However, the percentages of double/triple ovulation (80 %) was higher in the 0.2 mg EB treatment group. More importantly, the proportion of twin births (58 %) in the 0.2 mg EB treatment was also demonstrated to be the highest. 

### 3.4 Plasma estradiol concentration change among treatments

**Figure 3:**
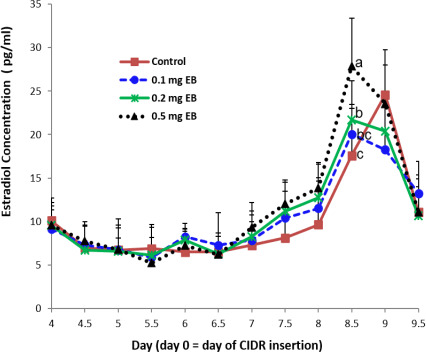
Dynamic change in the estradiol concentration in serum.

The E2 concentration did not differ among the four treatments on days 4, 4.5, 5, 5.5, 6, 6.5, and 9.5 (Fig. 3). In the experimental treatments, the concentration of E2 constantly increased from day 7 to day 8.5. On day 8.5, the concentration of E2 was at its highest, and the concentration in the 0.5 mg EB treatment was higher than that in the 0.2 mg or 0.1 mg EB treatments. The concentration of E2 declined from day 8.5 to day 9.5. However, in the control treatment, the concentration of E2 was at its highest on day 9 and quickly decreased to its lowest concentration on day 9.5.

## 4 Discussion

The present study investigated the impact of different doses of EB on the development and number of follicles after small doses of FSH. The most relevant observation from this work is that the 0.2 mg EB treatment increased the percentage of double/triple ovulation and twin birth in beef heifers. The follicular wave is characterized by a 2 or 3 d common-growth phase after follicle emergence at 4.0 mm in cattle (Bodensteiner et al., 1996; Ginther et al., 1997). When the largest follicle is about 8.5 mm, the first deviation occurs and the growth rate of the third-largest follicle decreases (Ginther et al., 1996, 1998; Wiltbank et al., 2000). The incorporation of estradiol benzoate into the CIDR protocol could enhance ovulation and pregnancy in dairy heifers (Mehmood et al., 2017). When a CIDR is inserted into cattle and progestin and oestrogen are injected, the concentrations of FSH and LH decrease to their lowest levels after 12 h (Cavalieri et al., 2003). Moreover, the development of antral follicles in ovaries is inhibited, and atresia occurs approximately 36 h after inserting the CIDR (Martínez et al., 2005). A new follicular wave begins 4 d later (Souza et al., 2009). In this study, four consecutive injections of low-dose FSH were administered to induce the formation of two dominant follicles in bovine ovaries during the expected restart of new follicular waves.

EB promoted a transient reduction in circulating FSH that initiated atresia in dominant ovarian follicles and delayed new follicular development (Elías et al., 1981; Burke et al., 2007). Spontaneous co-dominant follicles were associated with greater FSH and LH concentrations before deviation and a greater reduction in FSH after the beginning of deviation in cattle (Lopez et al., 2005b). The E2 concentration after deviation was higher for double-dominant compared with single-dominant heifers and cows (Lopez et al., 2005a). Oestrogen was administrated to inhibit the secretion of FSH via negative feedback (Gibbons et al., 1999; Ginther, 2000), resulting in the inhibition of non-dominant follicles' development after the first deviation. Therefore, in the present study, exogenous small doses of FSH were injected (from day 4.5 to day 6) before the first follicle deviation to induce the formation of co-dominant follicles. Following the 0.2 mg EB injections, the growth of other follicles was inhibited, and they became non-dominant follicles, thereby ensuring that the number (two or three) of dominant follicles did not increase. When 0.1 mg EB was injected, the dose was too small to inhibit the development of other follicles; thus the number of dominant follicles increased. However, an excessive dose of EB (0.5 mg EB) was found inhibit the development of co-dominant follicles, resulting in non-ovulation or single ovulation.

The results of the present work show that the size of the follicles was approximately equal to the theoretical value on day 4 (4.0 mm) and day 6.5 (8.5 mm). After EB injections, beginning on day 6.5, it was noted that the growth rate tended to decrease as the dose of EB increased, thereby showing that the size of the follicles was constrained by EB and that the degree of suppression was greater for larger doses of EB. As a result, the number of large follicles and the percentage of multiple ovulations decreased as the dose of EB increased. The number of ovulated follicles was higher than the number of fetuses (Fitzgerald et al., 2014). The reason for this was most likely fertilization failure (Echternkamp et al., 2009) or embryonic/fetal loss in the early stages of pregnancy (Silva-del-Rí et al., 2009). The results of the present study also revealed that the numbers of ovulated follicles and fetuses were inconsistent. Plasma concentrations of estradiol-17 β were elevated to 12 pg mL^−1^ during the initial 24 h following the intramuscular injection of 1 mg of EB, compared with a baseline of 1 pg mL^−1^ in untreated controls in non-lactating Friesian cows ( P<0.001 ) (Burke et al., 2000). Estradiol concentrations in circulation at day 0 were greater in EB-treated cows ( P=0.00 3) compared with those in the control group (Dysart et al., 2021). In the present study, the E2 concentration was higher in the experimental treatments than in the control before day 8, and the peak value also occurred 0.5 d earlier, thereby demonstrating that EB treatment could regulate E2 dynamic changes in serum during the development of follicles.

## 5 Conclusions

This work demonstrated that exogenous EB could modulate the development of two to three co-dominant follicles at the deviation stage after a small-dose FSH treatment; moreover, it could result in the production of two calves. The number of twin births was higher in animals treated with 0.2 mg EB than in the other treatments.

## Data Availability

The data sets used in this paper are available from the corresponding author upon request.
